# Novel Fig-Associated Viroid-Like RNAs Containing Hammerhead Ribozymes in Both Polarity Strands Identified by High-Throughput Sequencing

**DOI:** 10.3389/fmicb.2020.01903

**Published:** 2020-08-18

**Authors:** Alejandro Olmedo-Velarde, Beatriz Navarro, John S. Hu, Michael J. Melzer, Francesco Di Serio

**Affiliations:** ^1^Plant and Environmental Protection Sciences, University of Hawai‘i at Mānoa, Honolulu, HI, United States; ^2^Istituto per la Protezione Sostenibile delle Piante, Consiglio Nazionale delle Ricerche, Bari, Italy

**Keywords:** circular RNA, infectious RNAs, *Ficus carica*, next generation sequencing, non-coding RNAs, ribozyme, viroid, virusoid

## Abstract

Based on high-throughput sequencing (HTS) data, the existence of viroid-like RNAs (Vd-LRNAs) associated with fig trees grown in the Hawaiian Islands has been predicted. One of these RNAs has been characterized as a circular RNA ranging in size from 357 to 360 nucleotides. Structural and biochemical features of this RNA, tentatively named fig hammerhead viroid-like RNA (FHVd-LR), markedly resemble those previously reported for several viroids and viroid-like satellite RNAs (Vd-LsatRNAs), which are non-protein-coding RNAs infecting their hosts autonomously and in combination with a helper virus, respectively. The full-length sequence of FHVd-LR variants was determined by RT-PCR, cloning, and sequencing. Despite a low global sequence identity with known viroids and Vd-LsatRNAs, FHVd-LR contains a hammerhead ribozyme (HRz) in each polarity strand. Northern blot hybridization assays identified the circular and linear forms of both polarity strands of FHVd-LR and showed that one strand, assigned the (+) polarity, accumulates at higher levels than the (−) polarity strand *in vivo.* The (+) polarity RNA assumes a rod-like secondary structure of minimal free energy with the conserved domains of the HRzs located in opposition to each other, a feature typical of several viroids and Vd-LRNAs. The HRzs of both FHVd-LR polarity strands were shown to be active *in vitro* during transcription, self-cleaving the RNAs at the predicted sites. These data, together with the sequence variability observed in the cloned and sequenced full-length variants, indicate that FHVd-LR is a novel viroid or Vd-LsatRNA. According to HTS data, the coexistence of FHVd-LR of different sizes in the same host cannot be excluded. The relationships of FHVd-LR with previously reported viroids and Vd-LsatRNAs, and the need to perform bioassays to conclusively clarify the biological nature of this circular RNA, are discussed.

## Introduction

Viroids are small, infectious, non-protein-coding circular RNAs, so far identified only in plants (Kovalskaya and Hammond, 2014; Flores et al., 2015; Gago-Zachert, 2016). Other RNAs resembling viroids from a structural point of view, but differing from them at the biological level, or not conclusively characterized in this respect, have also been reported from plants and designated as viroid-like RNAs (Vd-LRNAs). Viroid-like satellite RNAs (Vd-LsatRNAs) and retroviroid-like elements are Vd-LRNAs markedly diverging from viroids at the biological level. While viroids replicate and systemically infect their host plants autonomously (in the absence of any helper virus), the infectivity of Vd-LsatRNAs depends on a coinfecting helper virus (Navarro et al., 2017), Vd-LsatRNAs are encapsidated by the helper virus capsid proteins and have been also named virusoids (Symons and Randles, 1999). Both viroids and Vd-LsatRNAs differ from carnation small Vd-LRNA, which is non-infectious and has a DNA counterpart integrated in the genome of a plant pararetrovirus (Daròs and Flores, 1995; Vera et al., 2000) or in the plant genome (Hegedus et al., 2004). For this reason, carnation small Vd-LRNA is considered a retroviroid-like element.

Viroids replicating in the nucleus and the chloroplast have been classified into the families *Pospiviroidae* and *Avsunviroidae*, respectively (Di Serio et al., 2014). Members of the two families also differ in structural and other functional features. In viroids of the family *Pospiviroidae*, one RNA polarity strand generates circular forms that assume a rod-like or quasi-rod-like secondary structure of minimal free energy containing a central conserved region (CCR) and other conserved motifs. The CCR is involved in the replication of nuclear viroids through an asymmetric rolling-circle mechanism (Flores et al., 2014). Viroids of the family *Avsunviroidae* lack the CCR and other typical structural motifs conserved in nuclear viroids. Instead, they assume rod-like, quasi-rod-like, or branched conformation and contain hammerhead ribozymes (HRzs) (Di Serio et al., 2018b). HRzs are inactive in the most stable viroid RNA conformation, but they assume an active structure responsible for RNA self-cleavage (without the catalytic contribution of any protein) during replication (Hutchins et al., 1986). In contrast to nuclear viroids, members of the family *Avsunviroidae* replicate through a symmetric rolling-circle mechanism, in which self-cleaved oligomeric RNAs of both polarity strands are circularized (Flores et al., 2014). HRzs with similar functional roles during replication also have been identified in several Vd-LsatRNAs (Navarro et al., 2017). However, in most of them the HRz is present in only one polarity, with the other polarity containing a different ribozyme (named paperclip) or no ribozyme at all. In accord with the presence of ribozymes in one or both polarity strands, asymmetric or symmetric rolling-circle replication mechanisms have been proposed for Vd-LsatRNAs, respectively (Navarro et al., 2017). Among Vd-LsatRNAs, only those of lucerne transient streak virus (LTSV, genus *Sobemovirus*), cereal yellow dwarf virus-RPV (genus *Polerovirus*), and two cherry small circular viroid-like RNAs (cscRNAs, likely associated with a mycovirus) contain two HRzs in both polarity strands (Di Serio et al., 1997, 2006; Navarro et al., 2017).

In tissues infected by viroids or Vd-LsatRNAs, the two polarity strands of the infectious RNAs are detectable, but generally one strand accumulates at a higher level and, by convention, is designated the (+) strand. Akin to viruses, viroids and Vd-LsatRNAs have the typical features of quasispecies (Codoñer et al., 2006; Flores et al., 2014), which is consistent with the observation that these infectious agents accumulate in their hosts as populations of closely related sequence variants, among which one (the master sequence) or a few may prevail depending on the selection pressures imposed by the host and environment (Di Serio et al., 2017).

Edible fig (*Ficus carica* L.) trees are known to be natural hosts of several viruses (Elbeaino et al., 2010, 2012; Laney et al., 2012; Ale-Agha and Rakhshandehroo, 2014; Minafra et al., 2017). Viroids of the family *Pospiviroidae*, including hop stunt viroid, citrus exocortis viroid, and a viroid resembling apple dimple fruit viroid, have also been reported in fig trees (Yakoubi et al., 2007; Chiumenti et al., 2014). The latter viroid was initially identified in fig by high-throughput sequencing (HTS), a powerful technology that has been applied to investigate the virome of several plant species (Hadidi et al., 2016; Villamor et al., 2019). Starting with HTS data, we report the identification of Vd-LRNAs containing HRzs in fig. One of these RNAs has been molecularly characterized, and its relationships with previously reported viroids and Vd-LsatRNAs are discussed.

## Materials and Methods

### Plant Material, RNA Isolation, and HTS

A fig plant from a commercial nursery on the island of Kauai, Hawaii, displaying symptoms of severe mosaic and leaf distortion, was analyzed by HTS. Nucleic acid preparations enriched in double-stranded RNAs (dsRNAs) were obtained from root tissue as described in Navarro and Di Serio (2018) and used as a source for generating a random-amplified cDNA library (Melzer et al., 2010). Sequencing of the cDNA library was performed using a 454 GS FLX Titanium platform (Roche, Branford, CT, United States) at the University of Hawaii’s Advanced Studies in Genomics, Proteomics and Bioinformatics (ASGPB) laboratory.

In 2018 and 2019, samples composed of mixed leaves, petioles, and green bark from twelve fig trees displaying symptoms resembling those of the Kauai fig sample were collected from three locations on the island of Oahu (Hawaii). Total nucleic acids were extracted as described by Li et al. (2008) and tested by RT-PCR (see below). Nucleic acids enriched in highly structured RNAs were obtained from young leaf, bark, and petiole tissues of the fig trees with buffer-saturated phenol and partitioning the nucleic acids by chromatography on non-ionic cellulose CF-11 (Whatman, Maidstone, United Kigdom) as described previously (Pallás et al., 1987).

### Bioinformatic Analysis

Analyses of HTS data were performed as described earlier (Olmedo-Velarde et al., 2019). Briefly, after trimming, quality control, and *de novo* assembly using Trinity (Grabherr et al., 2011), Velvet (Zerbino and Birney, 2008), and Unicycler (Wick et al., 2017), all the assembled contigs were sorted by length. Duplicate contigs and those <100 nucleotides (nt) were discarded using Geneious v.10.1.3^1^. The remaining contigs were screened for viral and viroid sequence homology using BlastX and BlastN, respectively^2^. Alignments of HTS reads with a sequence reference and reassembling of selected reads were performed using Bowtie (Langmead et al., 2009) and Phrap (Machado et al., 2011), respectively, and then implemented by the MacVector Assembler platform (17.5.3, MacVector, Inc., Apex, NC, United States). Multiple alignments of nucleotide sequences were performed using Clustal Omega (Sievers et al., 2011). RNAfold software (Lorenz et al., 2011) was used to predict the secondary structure of minimal free energy of the RNAs.

### RT-PCR and Cloning

Total nucleic acids were reverse transcribed using random hexamers and Superscript IV reverse transcriptase (Invitrogen, Thermo Fisher Scientific, Waltham, MA, United States), following the manufacturer’s instructions. Two microliters of the cDNA reaction served as template for PCR amplification using 0.4 units of Phusion High-Fidelity DNA polymerase (Thermo Fisher Scientific, Waltham, MA, United States) in a 20-μl mixture containing 1 × reaction buffer, 0.25 mM of dNTPs, and 0.5 μM of two FHVd-LR-specific primers (Table 1) and using the following cycling conditions: initial denaturation at 98°C for 30 s, followed by 33 cycles at 98°C for 15 s, 59°C for 15 s, 72°C for 15 s, and a final extension step at 72°C for 7 min. After agarose gel purification, an adenine-residue overhang was added at the 5′ end of the amplicons using GoTaq DNA polymerase (Promega, Madison, WI, United States). Amplification products of the expected size (monomeric and dimeric) were then cloned into a pGEMT-Easy vector (Promega, Madison, WI, United States) and sequenced by Sanger Sequencing Custom Service (Macrogen, Amsterdam, Netherlands).

**TABLE 1:** Primers used in this study.

**Name**	**Strand**	**Primer sequence (5′ to 3′)**	**Position**
FHVd-1F	(+)	CTCTGCCTGGAACGCTATGC	79–98
FHVd-2R	(−)	GAGCCGAAGAGGTGAGAGTC	79–60
FHVd-3F	(+)	GGAAAACACATTCCTAGACTTC	228–249
FHVd-4R	(−)	TACTGATGAGTCCAAAAGGACG	227–206
FHVd-5F	(+)	CTGATGAGAACAAAAGTTCGAAAC	19–42
FHVd-6R	(−)	TTGGATCACACAATCCAATACCTT	353–18
RACE 5′		CGCGGATCCCCCCCCCCCC	

### Northern Blot Hybridization

Nucleic acid preparations enriched in highly structured RNAs were subjected to double polyacrylamide gel electrophoresis (PAGE) (Flores et al., 1985). Briefly, the nucleic acids were separated by two consecutive 5% PAGE, the first under non-denaturing conditions (TAE buffer: 40 mM Tris, 20 mM sodium acetate, 1 mM EDTA, pH 7.2) and the second under denaturing conditions (8 M urea and 0.25 × TBE buffer). After staining the second gel with ethidium bromide, the nucleic acids were electroblotted to a nylon membrane (Hybond-N, Amersham, Little Chalfont, United Kingdom) in 0.5 × TBE buffer and immobilized by UV cross-linking. The membranes were then hybridized with DIG-labeled riboprobes complementary to (+) or (−) polarity strands of FHVd-LR as described previously (Hajizadeh et al., 2012). The hybridization signals were revealed with an anti-DIG alkaline phosphatase conjugate and the chemiluminescence substrate CSPD (Roche Applied Science) and visualized with a ChemiDoc Touch Imaging system (Bio-Rad, Hercules, CA, United States). The riboprobes were generated by *in vitro* transcription from linearized plasmids containing the full-length cDNA of FHVd-VL (see below) using a commercial Dig-labeling kit (Roche Diagnostics GmbH, Germany).

### Analyses of RNA Self-Cleavage

Monomeric and dimeric transcripts of both polarity strands were obtained by *in vitro* transcription of plasmids containing monomeric and head-to-tail dimeric FHVd-LR cDNA sequences in both orientations. Recombinant plasmids were linearized by digestion with the appropriate restriction enzyme (*Sal*I or *Nco*I) and, after phenol–chloroform extraction and ethanol precipitation, used as a template for the *in vitro* transcription with T7 or SP6 RNA polymerase (Forster et al., 1990). The transcription reactions containing the primary transcripts and their self-cleavage products were separated in 5% PAGE containing 8 M urea and 1 × TBE (89 mM Tris, 89 mM boric acid, 2.5 mM EDTA, pH 8.3), stained with ethidium bromide and UV visualized.

The 5′ terminal sequence of the 3′ self-cleavage product of the FHVd-LR monomeric transcripts of both polarity strands was determined by 5′ RACE experiments. Briefly, 3′ RNA fragments were eluted from the denaturing 5% PAGE by phenol–chloroform extraction and ethanol precipitation. The eluted RNA was reverse transcribed (as described above) using FHVd-4R and FHVd-5F (see Table 1) for the (+) and (−) polarity RNA fragment, respectively. Following the addition of a poly(dG) tail, the tailed cDNAs were PCR amplified using GoTaq DNA polymerase (Promega, Madison, WI, United States) with a 5′ RACE primer (Table 1) and the same primer used for the cDNA synthesis. PCR amplicons were gel-purified, cloned, and sequenced as reported above.

## Results

A cDNA library was generated from dsRNAs extracted from a fig tree with symptoms of mosaic and leaf distortion, grown on Kauai, Hawaii. HTS of such a library produced 262,700 reads with an average size of approximately 500 base pair (bp), which were filtered for quality and *de novo* assembled, generating 1,183 contigs (size 100 to 17,165 nt). BlastX and BlastN searches against the viral database revealed contigs with significant sequence identity to several viruses, including badnaviruses, closteroviruses, emaraviruses, endornaviruses, totiviruses, trichoviruses, and umbraviruses (Olmedo-Velarde et al., unpublished data). Moreover, BlastN analysis revealed two contigs of 462 and 674 nt that shared sequence similarity with some viroids and Vd-LRNAs. In particular, a short region of 40–50 nt of both contigs shared 84–87% sequence identity with HRzs contained in eggplant latent viroid (Fadda et al., 2003), grapevine hammerhead Vd-LRNA (Wu et al., 2012), and cscRNAs (Di Serio et al., 1997, 2006). Interestingly, each contig consisted of a partial direct repeat of a monomeric sequence of 358 and 390 nt, respectively (Supplementary Figure S1), thus suggesting a possible multimeric or circular nature of the corresponding RNAs. The monomeric sequences of the two contigs shared about 84% sequence identity with each other. More specifically, they shared almost identical sequences spanning a region of 260 nt, while the remaining part of the molecules (about 120 nt) largely diverged (Supplementary Figure S2). Despite the sequence diversity, the secondary structure of lowest free energy of both RNAs was of the rod-like class, with the largely divergent sequences located in the terminal right regions (Supplementary Figure S2). The two RNAs contained HRzs in both polarity strands (see below), thus displaying typical structural features previously reported for members of the family *Avsunviroidae* and in some Vd-LsatRNAs (Di Serio et al., 2018b; Navarro et al., 2017). Although these data strongly suggested the possible existence of at least two novel Vd-LRNAs in fig, it was not possible to confirm these preliminary results because the original fig source from Kauai was destroyed. In an attempt to identify additional isolates containing similar Vd-LRNAs, specific primers were designed to test other fig trees by RT-PCR.

### Identification of a Novel Fig Viroid-Like RNA on the Island of Oahu

**FIGURE 1:**
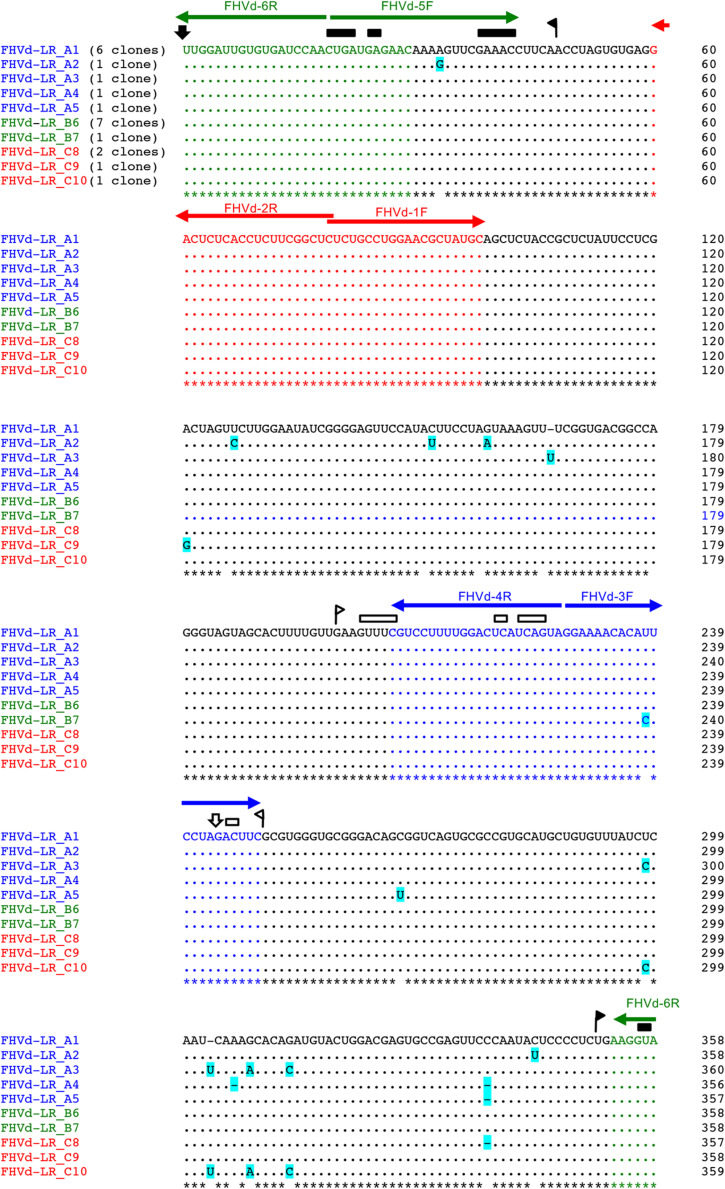
Multiple-sequence alignment of full-length cDNAs of fig hammerhead viroid-like RNA (FHVd-LR) variants amplified with primer pairs FHVd-1F/2R (in red), FHVd-3F/4R (in blue), and FHVd-5F/6R (in green). Names of the sequence variants and primers (horizontal arrows) used to amplify the corresponding cDNAs are reported with the same colors; the numbers of clones containing the same variant are indicated in brackets. The reference variant (FHVd-LR_A1) reported at the top is identical to the variant FHVd-LR_B6 and, being the most frequently sequenced (found in 13 independent clones), also corresponds to the master sequence in the population. Nucleotide identity and gaps with respect to the FHVd-LR_A1 variant are indicated by dots and dashes, respectively. The light blue background highlights mutations. Regions involved in the formation of (+) and (−) hammerhead structures are delimited by flags; arrows and bars indicate the self-cleavage sites and the nucleotides conserved in most natural hammerhead structures, respectively; filled (black) and open symbols refer to (+) and (−) polarity, respectively. Nucleotide positions in the multiple alignments are reported on the left.

An RT-PCR-based preliminary survey was performed on the island of Oahu, Hawaii, using primers FHVd-1F and FHVd-2R (Table 1) derived from two adjacent regions of a sequence common to the two contigs and, therefore, expected to amplify full-length cDNAs of the potential circular RNAs (Candresse et al., 1998). Three out of the twelve fig trees examined, all grown in the same location on Oahu, generated amplification products of about 360 bp. Cloning and sequencing of the amplicons showed sequence variants ranging in size from 357 to 359 nt, which differed from each other in a few positions (Figure 1), The conserved nucleotides reported in almost all natural HRzs were found in both polarity strands of the cloned RNA variants (Figure 1), and BlastN searches confirmed that only the short-sequence fragment corresponding to the ribozyme domain matched with some members of the family *Avsunviroidae* and with csc-RNAs. Importantly, the sequenced variants from Oahu and the short Vd-LRNA from the fig tree on the island of Kauai shared high identity (96%), with only 14 polymorphic positions observed in a pairwise alignment between them (Supplementary Figure S3). Altogether, these data suggested that the RNA identified from Oahu was closely related to those found by HTS in the Kauai sample and could represent a novel Vd-LRNA, hereafter named fig hammerhead viroid-like RNA (FHVd-LR).

### Primary and Proposed Secondary Structure of FHVd-LR

**FIGURE 2:**
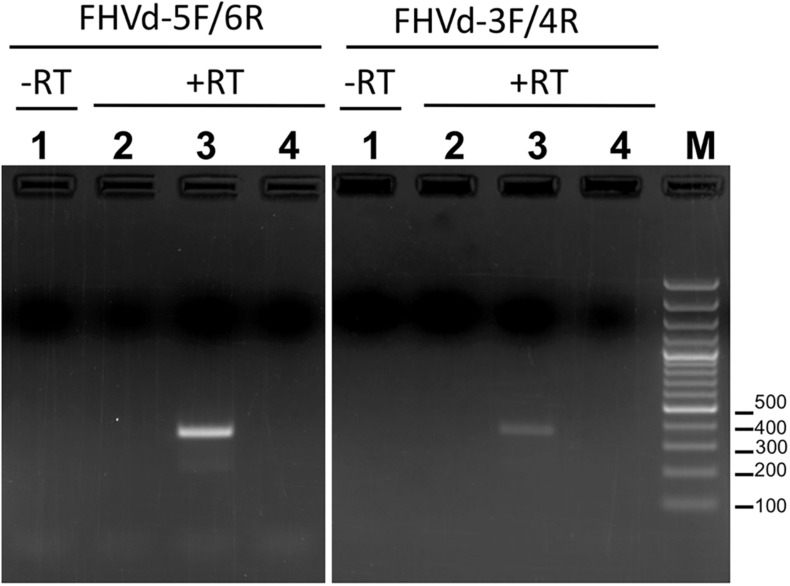
Amplification assays using RNA preparations from FHVd-LR-positive and -negative samples, and primer pairs FHVd-5F/6R (left) or FHVd-3F/4R (right). Lane 1, amplification was performed in the absence of reverse transcription (−RT) using an FHVd-LR-positive sample. Lanes 2 and 3, amplifications were performed after reverse transcription (+RT) using RNA preparations from FHVd-LR-negative or -positive samples, respectively. Lane 4 is a non-template control. The same FHVd-LR-positive sample was tested in lanes 1 and 3.

To further assess the nucleotide sequence composition in the region covered by the first primer set and the possible circularity of the RNA, full-length cDNAs were also amplified and cloned using two additional pairs of adjacent primers (FHVd-3F/4R and FHVd-5F/6R, Table 1). Sequencing of 10 and 8 clones of the RT-PCR amplicons generated with the two respective primer sets revealed variants ranging in size from 356 to 360 nt. They showed high sequence identity (97.8–100%) between them and to those obtained previously using the primer set FHVd-1F/2R. A total of nine different sequence variants were annotated in GenBank (with the accession numbers from MT57734 to MT577542). The most frequently sequenced variant was FHVd-LR_A1, which differed from the others in up to six positions and will be considered as the reference variant for this new Vd-LRNA (Figure 1). These results support the circular and quasispecies nature of the RNA. Multiple-sequence alignments also revealed the absence of nucleotide variability in the region covered by FHVd-1F/2R, while only two polymorphic positions were found in the regions targeted by the other two primer pairs (FHVd-3F/4R and FHVd-5F/6R). Interestingly, 9 out of 15 changes mapped in a region covering almost 100 nt, between positions 265 and 358 of the multiple sequence alignment (Figure 1). FHVd-LR was composed of 22.6, 28.2, 24.6, and 24.6% of A, U, C, and G, respectively. Therefore, its G + C content (49.2%) resembled that of most viroids (ranging from 52.2% to 61.6%) and Vd-LsatRNAs (ranging from 50.0 to 63.6%). However, the G + C content differed from avocado sunblotch viroid (ASBVd) (Symons, 1981), which is unique in its low G + C content of about 38%. The existence of a DNA counterpart of FHVd-LR, and therefore its potential retroviroid-like nature, was excluded based on the negative results of amplification by PCR without previous reverse transcription using nucleic acid preparations from a FHVd-LR-positive tree and different primer pairs (Figure 2).

**FIGURE 3:**
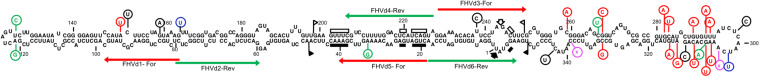
Primary and predicted secondary structures of lowest free energy of the fig hammerhead viroid-like RNA variant A1 (FHVd-LR_A1) collected from the island of Oahu. Regions involved in the formation of (+) and (−) hammerhead structures are delimited by flags; arrows and bars indicate the self-cleavage sites and the nucleotides conserved in most natural hammerhead structures, respectively; filled (black) and open symbols refer to (+) and (−) polarity, respectively. Mutated positions in other FHVd-LR variants are indicated in circles, with compensatory mutations, insertions, and deletions reported in green, blue, and pink, respectively. Nucleotide changes with respect to the short VL-RNA from the island of Kauai are in red. Horizontal arrows delimit the regions targeted by primer pairs used in the amplification steps of the cloning and sequencing strategy.

When the secondary structure of minimal free energy was calculated, FHVd-LR variants assumed a rod-like conformation with about 70% paired residues (Figure 3). In such a structure, the conserved sequences in the natural HRzs were located in a central region and opposite to each other (Figure 3), as previously observed in most members of the family *Avsunviroidae* and several Vd-LsatRNAs. The proposed rod-like secondary structure of FHVd-LR was also supported by most of the heterogeneity found in the sequenced variants consisting of changes in loops or compensatory mutations, which did not result in major modifications of the proposed conformation (Figure 3). It is interesting that the nucleotide changes were asymmetrically distributed in the proposed structure, with most of them mapping at the terminal right domain of the molecule and covering about 120 nt.

As reported above, FHVd-LR and the short Vd-LRNA from Kauai shared high sequence identity. Interestingly, most nucleotide changes in the latter RNA mapped in the terminal right domain of the secondary structure proposed for FHVd-LR and were compensatory mutations or covariations preserving the rod-like conformation (Figure 3). These findings highlighted an additional structural parallelism between the variants from the Oahu samples, which we confirmed by cloning and sequencing, and the Vd-LRNAs from the Kauai isolate that were detected only *in silico* due to destruction of the original tree.

### Circularity and Accumulation Levels of Both Polarity Strands of FHVd-LR

**FIGURE 4:**
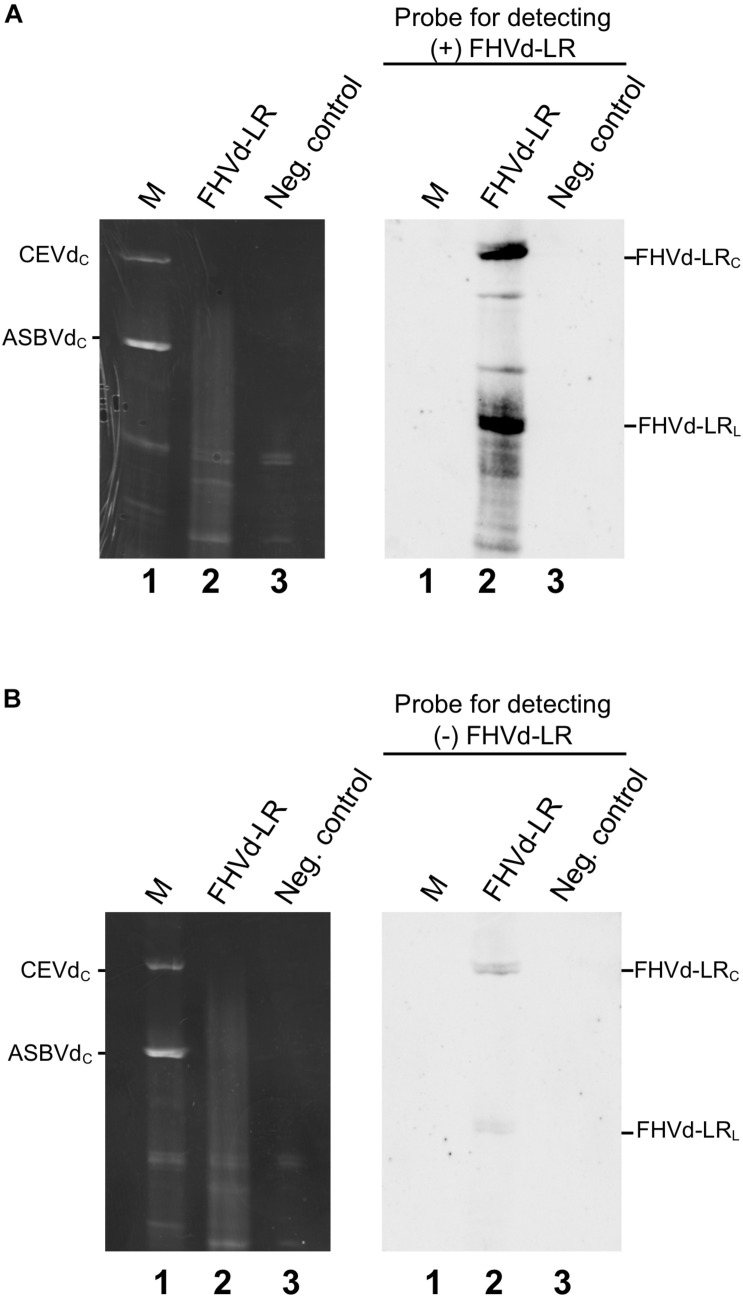
Northern blot hybridization assays to detect circular RNAs and identify the most abundant FHVd-LR strand denoted (+) polarity strand. Fig RNA preparations separated by double PAGE (left) were transferred to nylon membranes and hybridized with equalized full-length digoxigenin-labeled riboprobes for detecting the (+) and (−) FHVd-LR strands **(A,B)**, respectively. The second (denaturing) gel of the double PAGE, stained with ethidium bromide, and the northern blot results are respectively reported on the left and on the right of each panel. Lane 1 corresponds to RNA preparations from *Gynura aurantiaca* and avocado infected by citrus exocortis viroid (CEVd, 371 nt) and avocado sunblotch viroid (ASBVd, 246 nt) respectively, used as molecular markers (M); lane 2 is an RNA preparation from a fig tree that tested positive to FHVd-LR by RT-PCR assay; lane 3 is an RNA preparation from FHVd-LR fig tree that tested negative to FHVd-LR and used as negative control. Identical aliquots of the same RNA preparations were loaded in the gels shown in panels **(A,B)**. The positions of circular forms of CEVd and ASBVd (CEVdc and ASBVdc), visible in the gel stained with ethidium bromide, are reported on the left; the positions of circular and linear forms of FHVd-LR detected by the specific probes for the (+) and (−) polarity strands [panels **(A,B)**, respectively] are indicated on the right. Bands between the circular and linear forms could correspond to artifacts generated by UV-induced cross-links during the visualization of the first non-denaturing gel of the double PAGE, as observed previously for some viroids (Hernández et al., 2006; Serra et al., 2018).

The presence of FHVd-LR in fig and evidence of its circularity were further inferred by northern blot hybridization. Nucleic acid preparations enriched in highly structured RNAs from fig trees that tested positive and negative in the RT-PCR assays were loaded side by side in a non-denaturing PAGE followed by a denaturing PAGE (double PAGE), a method specifically developed to separate circular from linear RNAs of the same size (Flores et al., 1985). Moreover, to test whether both polarity strands of FHVd-LR generated circular forms and to ascertain whether one of them accumulated at a higher level *in vivo*, equalized riboprobes specific for detecting each FHVd-LR polarity strand were used separately in parallel hybridization experiments. As expected, the circular and linear forms of FHVd-LR were only detected in the samples from the fig trees that previously tested positive by RT-PCR, thus confirming the association of this RNA with some fig plants only (Figure 4). Circular forms of both FHVd-LR polarity strands were detected. Based on the intensity of the hybridization signals, one polarity strand accumulated at higher level *in vivo* (Figure 4). Therefore, this strand, corresponding to the sequences in Figures 1, 2, was considered as the (+) FHVd-LR polarity. The possibility that one probe could cross-hybridize with the strand of the same polarity due to the self-complementarity of the FHVd-LR sequence was excluded by northern blot assays showing that this did not happen under the experimental conditions used (Supplementary Figure S4).

### Hammerhead Ribozymes of FHVd-LR Are Active During Transcription

**FIGURE 5:**
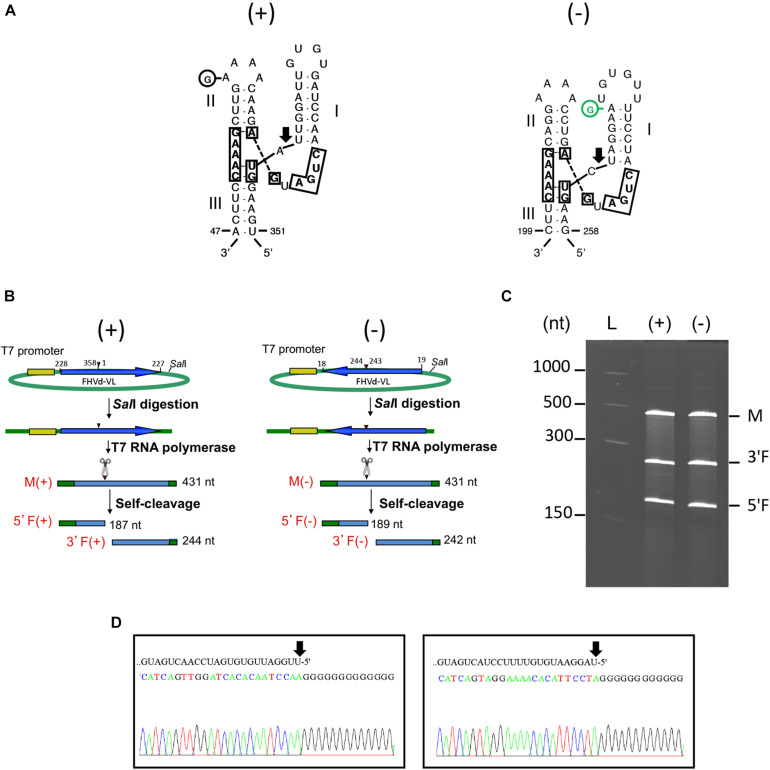
**(A)** Primary and Y-shaped secondary structure of hammerhead ribozymes (HRzs) of (+) and (−) FHVd-LR are presented considering the existing X-ray crystallography data on this class of ribozymes (Pley et al., 1994). Stems I and II are closed by loops. The nucleotides of the catalytic core conserved in most natural hammerhead structures are boxed. The cleavage site of each ribozyme is indicated by an arrow. Nucleotides in the (+) and (−) polarity are numerated considering their positions in the variant FHVd-LR_A1 (+). Mutations are indicated in circles, with the compensatory mutation reported in green. **(B)** Schematic representation of the DNA templates and monomeric RNA products generated by *in vitro* transcription. Plasmids containing the monomeric sequence of FHVd-LR in opposite orientations were linearized with *Sal*I and transcribed with T7 RNA polymerase to produce monomeric transcripts (M) of opposite polarity and the respective 5′ and 3′ fragments (5′F and 3′F, respectively) derived from the HRz self-cleaving activity. In green, plasmid sequences; in yellow, polymerase promoter; in blue, FHVd-LR sequence, with the arrows indicating the (+) orientation; arrowheads and scissors mark the position of the self-cleavage sites. Numbers on the right of each RNA fragment indicate its expected size. **(C)** Analyses by PAGE of the *in vitro* transcription of the plasmids containing (+) and (−) monomeric FHVd-LR cDNA. L, RNA ladder with sizes indicated on the left; see panel **(B)** for M, 3′F and 5′F abbreviations. **(D)** Determination of self-cleavage site by 5′ RACE of 3′F fragment resulting from the HRz-mediated cleavage of FHVd-LR monomeric transcripts [generated as reported in panels **(B,C)**]. Sequencing electropherograms of 5′ RACE products of the (+) and (−) 3′F fragments are shown on the left and right, respectively, with the self-cleaved RNA sequence reported on the top and the 5′ terminal nucleotide indicated by the arrow.

Fig hammerhead viroid-like RNA HRzs of both polarity strands were composed of three hairpins, two of them closed by short loops, which are located around the central core containing the predicted self-cleavage site (Figure 5A). In the (−) HRz, this site is preceded by a GUC trinucleotide, as in most HRzs of other viroids and Vd-LRNAs (Hutchins et al., 1986; Navarro et al., 2017). In contrast, a GUA trinucleotide was found at the same position in the (+) HRz of FHVd-LR, a situation previously reported only in the (−) HRz of the Vd-LsatRNAs of velvet tobacco mottle virus and LTSV (Forster et al., 1988; Collins et al., 1998) and in the (+) HRz of cscRNAs (Di Serio et al., 2006). The relevance *in vivo* of the FHVd-LR HRzs was confirmed by the limited sequence variability observed in these catalytic domains in the FHVd-LR variants (Figures 1, 3). Indeed, the single mutation detected in the (+) HRz mapped in the loop that closes the hairpin II, while that found in the (−) HRz was a compensatory mutation located in the stem II (Figure 5A). Therefore, both mutations preserved the typical hammerhead structures.

*In vitro* transcription of recombinant plasmids containing monomeric and dimeric head-to-tail constructs of FHVd-LR indicated that HRzs of both polarity strands were active, generating RNA fragments of sizes consistent with those expected and in accord with the self-cleavage of transcripts at the predicted sites (Figures 5B,C and Supplementary Figure S5). Moreover, the self-cleavage site of both polarity strands was further confirmed by 5′ RACE experiments, showing that the 3′ fragments generated by the ribozymes in each polarity strand had the expected 5′ terminal termini (Figure 5D).

## Discussion

Analyses of HTS data from a library prepared from dsRNAs allowed us to predict the existence of two novel Vd-LRNAs in a fig tree grown on the island of Kauai, Hawaii. The two potential small circular RNAs contained HRzs in both polarity strands and had closely related sequences of 358 nt and 390 nt that mainly diverged in a specific region of about 120 nt. Discrimination between the long and short Vd-LRNAs *in silico* was possible due to the availability of long reads, up to 382 nt, which covered common and divergent sequences of the two *de novo* assembled contigs. This finding suggests that HTS technologies providing long sequence reads, like the 454 platform used in the present study, increase the chances of detecting coexisting but slightly divergent sequence variants of Vd-LRNAs.

In the absence of additional material from the original plant isolate, *in silico* data were used to design specific primers and perform a preliminary RT-PCR survey of figs on the island of Oahu, Hawaii. This analysis resulted in the identification of several fig trees associated with FHVd-LR, a small circular RNA with HRz in both polarity strands closely related to the Vd-LRNAs from Kauai. The presence of FHVd-LR only in some fig trees from Oahu, but not in others, was confirmed by northern blot hybridization. Northern blotting also showed that linear and circular forms of both polarity strands of this RNA exist *in vivo*, with one strand accumulating at a higher level than the other. Further experiments showed that this RNA has no DNA counterpart and that it accumulates in infected tissues as a population of sequence variants differing from each other in a few positions. These are typical features of quasispecies previously reported for other RNA replicons, including viruses, viroids, and VL-sat RNAs (Biebricher and Eigen, 2006; Codoñer et al., 2006; Flores et al., 2014).

Hammerhead ribozyme-mediated self-cleavage of both polarity strands of FHVd-LR during transcription was demonstrated and correspondence between the predicted and actual self-cleavage sites confirmed by RACE experiments. Interestingly, the nucleotide changes observed in the HRzs of both polarity strands of FHVd-LR did not impair the formation of the stable hammerhead structures, thus upholding the relevance *in vivo* of these ribozymes. Altogether, these data strongly support that FHVd-LR is a novel Vd-LRNA that likely replicates through a symmetric rolling-circle mechanism as previously proposed for some viroids and Vd-LsatRNAs (Flores et al., 2011). However, these structural and biochemical features did not provide any indication on whether FHVd-LR is a viroid or a Vd-LsatRNA. Indeed, HRzs in both polarity strands have been reported in three Vd-LsatRNAs and in all viroids classified in the family *Avsunviroidae.* HRzs were also found in both polarity strands of two Vd-LRNAs whose nature remains unclear (grapevine hammerhead Vd-LRNA and cscRNAs, which possibly are a viroid and two Vd-LsatRNAs of a mycovirus, respectively) (Alioto et al., 2003; Wu et al., 2012; Minoia et al., 2014).

Fig hammerhead viroid-like RNA assumes a rod-like conformation of minimal free energy, the relevance *in vivo* of which is supported by the variability observed in the FHVd-LR population accumulating in fig tissues. Indeed, most nucleotide changes detected in the FHVd-LR sequence variants mapped at loops or were compensatory mutations or covariations preserving the proposed structure. Rod-like structures have been proposed for most viroids and Vd-LsatRNAs. In members of the family *Avsunviroidae*, the G + C content, morphology, and thermodynamic stability of the HRzs and the secondary structure are of taxonomic relevance (Di Serio et al., 2018b). When these structural elements are taken into consideration, FHVd-LR seems to diverge from all members of the family *Avsunviroidae*. Among them, only ASBVd adopts a rod-like conformation *in silico*, *in vitro*, and *in vivo* (Symons, 1981; López-Carrasco and Flores, 2017). However, with a 38% G + C content and thermodynamically unstable HRzs due to a short stem III (Forster et al., 1988), this viroid appears different from FHVd-LR, which has about 50% G + C and stable HRzs. In contrast, the remaining members of the family *Avsunviroidae*, which share with FHVd-LR a high G + C content and stable HRzs (Di Serio et al., 2018b; Serra et al., 2018), assume branched or bifurcated conformations. Actually, among all known Vd-LRNAs, only the Vd-LsatRNA of LTSV and the two Vd-LRNAs from cherry (cscRNAs) resemble FHVd-LR in their rod-like secondary structure, high G + C content (about 50%), and stable HRzs in both polarity strands (Forster and Symons, 1990; Di Serio et al., 2006). Additionally, akin to the HRzs of FHVd-LR, the (+) and (−) HRzs of the two cscRNAs and the (−) HRz of VdL-satRNA of LTSV also contain an atypical adenine residue preceding the self-cleavage site.

In this context, note that the *in silico* data reported here supported the coexistence of at least two Vd-LRNAs of different size in the fig sample from Kauai, a hypothesis that was not conclusively tested due to the destruction of the original isolate. Attempted amplifications by RT-PCR, using different primer pairs, of Vd-LRNAs larger than FHVd-LR in the Oahu isolates have so far been unsuccessful, suggesting the absence of larger coinfecting Vd-LRNAs. Moreover, the two cscRNAs coinfecting cherry trees were not simultaneously observed in all the studied isolates, with some of them being infected by only one (Minoia et al., 2014).

Although the FHVd-LR structural features are closer to the VL-satRNA of LTSV and to cscRNAs than to viroids, we are unable to predict the biological nature of this novel Vd-LRNA from fig. Only bioassays may answer the specific question of whether FHVd-LR is able to replicate autonomously and systemically infect fig (Di Serio et al., 2018a). In this respect, experiments to test the transmission of FHVd-LR to virus-free fig plants by grafting and slash-inoculation of RNA preparations enriched in Vd-LRNAs and of *in vitro*-generated dimeric head-to tail transcripts are ongoing. Slash-inoculated plants were negative by RT-PCR and northern blot assays one and two months post-inoculation, but such bioassays in woody hosts may require longer times for systemic infection (Di Serio et al., 2018a).

Although all viruses identified by HTS as potentially infecting the original fig tree from Kauai belong to viral taxa not containing helper viruses of Vd-LsatRNAs, the association of FHVd-LR with a plant virus (known or unknown) cannot be ruled out; further studies are needed. The association of FHVd-LR with a virus infecting a fungus or another organism associated with fig cannot be excluded either. It has been in fact reported that HTS applied to cDNA libraries from plant tissues may detect viruses (and possibly Vd-LRNAs) infecting other transiently plant-associated organisms (Hily et al., 2018).

## Conclusion

In conclusion, HTS allowed the initial identification of two novel Vd-LRNAs associated with fig, providing the necessary information for molecular studies to confirm the existence, self-cleaving activity mediated by HRzs, circularity of both polarity strands, and quasispecies nature of FHVd-LR. The data reported here make available detection methods to further investigate the biology and epidemiology of this and possibly other coinfecting novel Vd-LRNAs in fig trees.

## Data Availability Statement

The datasets presented in this study can be found in NCBI database GenBank, accession numbers from MT57734 to MT577542.

## Author Contributions

FD and MM supervised the project. AO-V and BN performed the experiments. AO-V, MM, JH, FD, and BN conceived the study and analyzed the data. FD, AO-V, and BN wrote the manuscript. All authors revised the manuscript.

## Conflict of Interest

The authors declare that the research was conducted in the absence of any commercial or financial relationships that could be construed as a potential conflict of interest.

## Footnotes

1 https://www.geneious.com

2 http://www.ncbi.nlm.nih.gov/

## Supplementary Material

FIGURE S1 Contigs of different size consisting of direct repeats (green arrows) generated by overlapping reads (in pink) obtained by HTS of a cDNA library generated from dsRNA extracted from a fig tree grown on the island of Kauai, Hawaii.

FIGURE S2 **(A)** Pairwise comparison between a short and a long viroid-like RNA (Vd-LRNA) identified *in silico* in a fig sample from the island of Kauai. Positions with identical nucleotides are denoted by asterisks. **(B)** Primary and predicted secondary structure of the free energy of the long and short Vd-LRNA variants from Kauai. Differences between both RNA molecules are denoted in red. Regions involved in the formation of (+) and (−) hammerhead structures are delimited by flags; arrows and bars indicate the self-cleavage sites and the nucleotides conserved in most natural hammerhead structures, respectively; filled (black) and open symbols refer to (+) and (−) polarity, respectively.

FIGURE S3 Pairwise comparison between the short viroid-like RNA (Vd-LRNA) from island of Kauai and FHVd-LR (master sequence) from the island of Oahu. The sequence identity is 96.1%. Positions with identical nucleotides are denoted by asterisks.

FIGURE S4 Northern blot hybridization with equalized full-length digoxigenine-labeled riboprobes for detecting FHVd-LR (+) or (−) strands (left and right panel, respectively). In each panel, lanes 1 and 2 correspond to equal amounts of dimeric FHVd-LR (+) and (−) transcripts, respectively. Positions of the FHVd-LR (+) and (−) linear monomeric forms (m) are indicated by an arrow. Position and size (in nt) of the RNAs used as molecular markers are reported on the left.

FIGURE S5 **(A)** Schematic representation of plasmids containing head-to-tail dimeric constructs of FHVd-LR and of the products generated by *in vitro* transcription. Plasmids containing the dimeric sequence of FHVd-LR in opposite orientations were linearized with *Sal*I or *Nco*I and transcribed with T7 or SP6 RNA polymerase, respectively. Transcription of these templates is expected to produce complete dimeric transcripts (D), fragments longer than a monomer (5′F-M and 3′F-M) generated by the self-cleavage of one hammerhead ribozyme (HRz), monomeric RNAs (M) and fragments (5′F and 3′F) generated by the self-cleavage of both HRzs. In green, plasmid sequences; in yellow, polymerase promoter; in blue, FHVd-LR sequence, with the arrows indicating the (+) orientation; arrowheads and scissors mark the positions of the self-cleavage sites. Numbers on the left of each RNA fragment indicate its expected size. **(B)** Analysis by PAGE of the *in vitro* transcription of plasmids containing (+) and (−) dimeric FHVd-LR cDNA; L1 and L2, RNA ladders with sizes indicated on the left and on the right, respectively; for other symbols see panel **(A)**.
