# Supplementary material for: Novel Fig-Associated Viroid-Like RNAs Containing Hammerhead Ribozymes in Both Polarity Strands Identified by High-Throughput Sequencing
Source: Front Microbiol. 2020 Aug 18;11:1903. doi: 10.3389/fmicb.2020.01903 (PMC7461866; doi:10.3389/fmicb.2020.01903)
Supplement: FIGURE S1 — Contigs of different size consisting of direct repeats (green arrows) generated by overlapping reads (in pink) obtained by HTS of a cDNA library generated from dsRNA extracted from a fig tree grown on the island of Kauai, Hawaii. [file Data_Sheet_1.PDF]

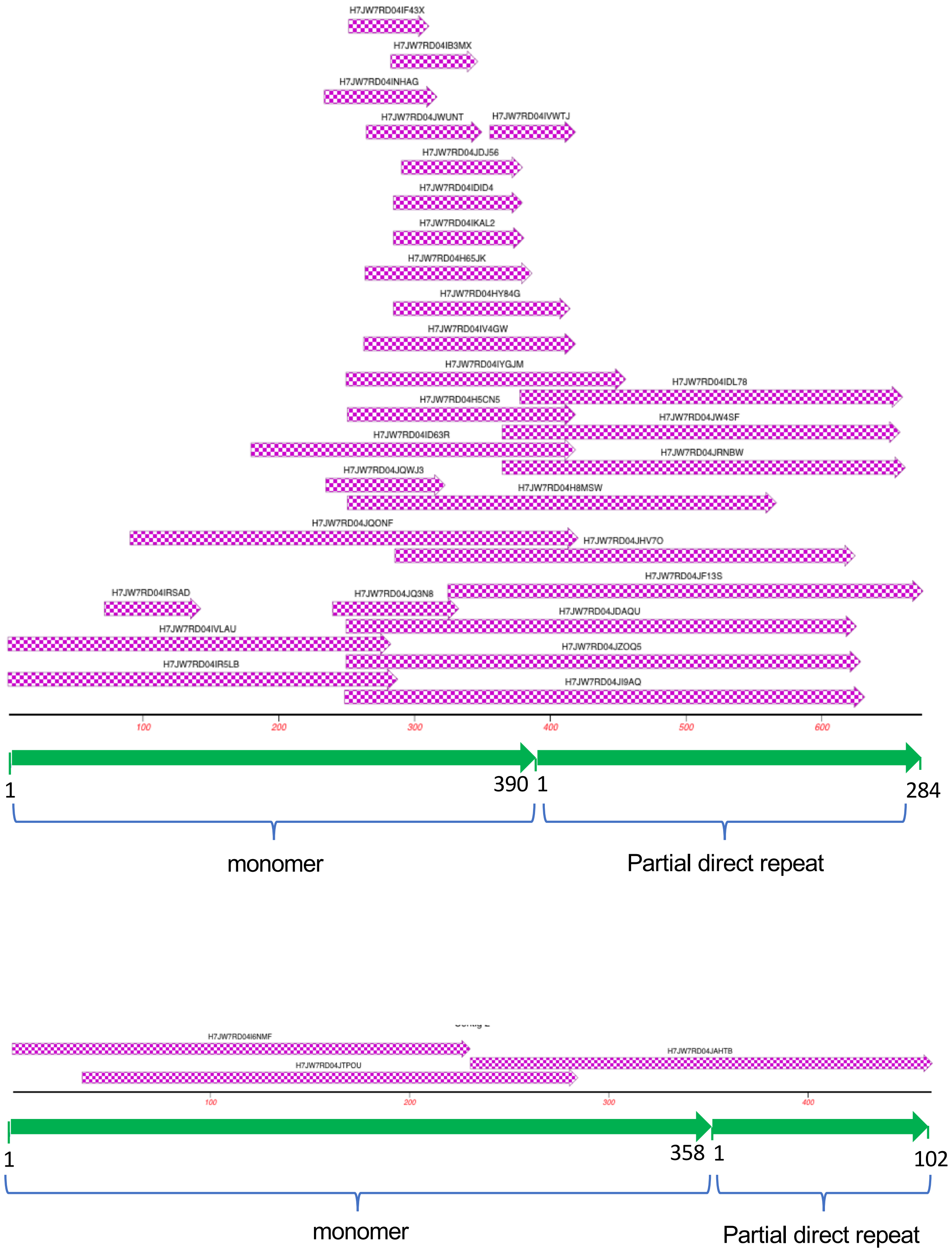

Figure S1 Contigs of different size consisting of direct repeats (green arrows) generated by overlapping reads (in pink) obtained by HTS of a cDNA library generated from dsRNA extracted from a fig tree grown on the island of Kauai, Hawaii.
