# Supplementary material for: Novel Fig-Associated Viroid-Like RNAs Containing Hammerhead Ribozymes in Both Polarity Strands Identified by High-Throughput Sequencing
Source: Front Microbiol. 2020 Aug 18;11:1903. doi: 10.3389/fmicb.2020.01903 (PMC7461866; doi:10.3389/fmicb.2020.01903)
Supplement: FIGURE S2 — (A) Pairwise comparison between a short and a long viroid-like RNA (Vd-LRNA) identified in silico in a fig sample from the island of Kauai. Positions with identical nucleotides are denoted by asterisks. (B) Primary and predicted secondary structure of the free energy of the long and short Vd-LRNA variants from Kauai. Differences between both RNA molecules are denoted in red. Regions involved in the formation of (+) and (−) hammerhead structures are delimited by flags; arrows and bars indicate the self-cleavage sites and the nucleotides conserved in most natural hammerhead structures, respectively; filled (black) and open symbols refer to (+) and (−) polarity, respectively. [file Data_Sheet_2.PDF]

A

```
Vd-LRNA_Kauai_long       TTGGATTGTGTGATCCAACTGACGAGAACAAAAGTTCGAAACCTTCAACCTAGTGTGAGG 60
Vd-LRNA_Kauai_short      TTGGATTGTGTGATCCAACTGATGAGAACAAAAGTTCGAAACCTTCAACCTAGTGTGAGG 60
                         ********************** *************************************

Vd-LRNA_Kauai_long       ACTCTCACCTCTTCGGCTCTCTGCCTGGAACGCTATGCAGCTCTACCGCTCTATTCCTCG 120
Vd-LRNA_Kauai_short      ACTCTCACCTCTTCGGCTCTCTGCCTGGAACGCTATGCAGCTCTACCGCTCTATTCCTCG 120
                         ************************************************************

Vd-LRNA_Kauai_long       ACTAGTTCTTGGAGTATCAAGGGAGTTCCATACTTCCTAGTAGAGTTTCGGTGACGGCCA 180
Vd-LRNA_Kauai_short      ACTAGTTCTTGGAATATCG-GGGAGTTCCATTCTTCCTAGTAAAGTTTCGGTGACGGCCA 179
                         ************* ****  *********** ********** *****************

Vd-LRNA_Kauai_long       GGGTAGTAGCACTTTTGTTGTAGAAGTTTCGTCCTTTTGGACTCATCAGTAGGAAAACAC 240
Vd-LRNA_Kauai_short      GGGTAGTAGCACTTTTGTT---GAAGTTTCGTCCTTTTGGACTCATCAGTAGGAAAACAC 236
                         *******************   **************************************

Vd-LRNA_Kauai_long       ATTCCTAGACTTCTGGTAGGTCTATACCATAGAATTTGAAACTTAACACTGGTGTGTCAA 300
Vd-LRNA_Kauai_short      ATTCCTAGACTTCGCGTGGGTGAGGGACA-----------GCCGTCAGTGCGCCGTATA 284
                         *************  ** ***      **                 ** **    **  *

Vd-LRNA_Kauai_long       G--GATCCACGTCTCTC-CCATTCTTAGACGTCGTTCCTGACGAAGAAGTCTGGTGAGTT 357
Vd-LRNA_Kauai_short      TGCTATGAATATCTCAATCAATTCATAGGTATA---CTGGACGA------GTGCGGAGTT 335
                             **  *  ****   * **** ***   *    *  *****       **  *****

VL_RNA_Kauai_long        GTGTTCTATGACGAAACCGACCTTCTGAAGGTA   390
Vd-LRNA_Kauai_short      ----------CCCAATACTCCCCTCTGAAGGTA   358
                                    * **  *  ** **********
```

B

Fig Vd-LRNA long

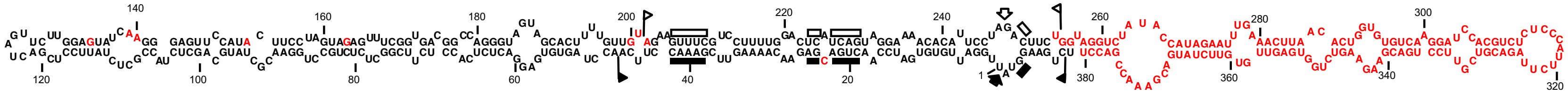

Fig Vd-LRNA short

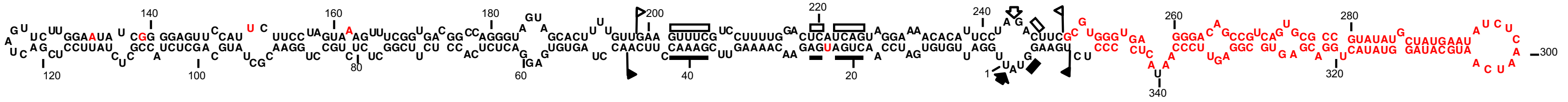

Figure S2. (A) Pairwise comparison between a short and a long viroid-like RNA (Vd-LRNA) identified *in silico* in a fig sample from the island of Kauai. Positions with identical nucleotides are denoted by asterisks. **(B)** Primary and predicted secondary structure of the lowest free energy of the long and short Vd-LRNA variants from Kauai. Differences between both RNA molecules are denoted in red. Regions involved in the formation of (+) and (-) hammerhead structures are delimited by flags; arrows and bars indicate the self-cleavage sites and the nucleotides conserved in most natural hammerhead structures, respectively; filled (black) and open symbols refer to (+) and (-) polarity, respectively.
