# Supplementary material for: Novel Fig-Associated Viroid-Like RNAs Containing Hammerhead Ribozymes in Both Polarity Strands Identified by High-Throughput Sequencing
Source: Front Microbiol. 2020 Aug 18;11:1903. doi: 10.3389/fmicb.2020.01903 (PMC7461866; doi:10.3389/fmicb.2020.01903)
Supplement: FIGURE S3 — Pairwise comparison between the short viroid-like RNA (Vd-LRNA) from island of Kauai and FHVd-LR (master sequence) from the island of Oahu. The sequence identity is 96.1%. Positions with identical nucleotides are denoted by asterisks. [file Data_Sheet_3.PDF]

```
Vd-LRNA Kauai short   TTGGATTGTGTGATCCAACTGATGAGAACAAAAGTTCGAAACCTTCAACCTAGTGTGAGG   60
FHVd-LR Oahu          TTGGATTGTGTGATCCAACTGATGAGAACAAAAGTTCGAAACCTTCAACCTAGTGTGAGG   60
                      ************************************************************

Vd-LRNA kAUAI short   ACTCTCACCTCTTCGGCTCTCTGCCTGGAACGCTATGCAGCTCTACCGCTCTATTCCTCG   120
FHVd-LR Oahu          ACTCTCACCTCTTCGGCTCTCTGCCTGGAACGCTATGCAGCTCTACCGCTCTATTCCTCG   120
                      ************************************************************

Vd-LRNA Kauai short   ACTAGTTCTTGGAATATCGGGGAGTTCCATTCTTCCTAGTAAAGTTTCGGTGACGGCCAG   180
FHVd-LR Oahu          ACTAGTTCTTGGAATATCGGGGAGTTCCATACTTCCTAGTAAAGTTTCGGTGACGGCCAG   180
                      ****************************** *****************************

Vd-LRNA Kauai short   GGTAGTAGCACTTTTGTTGAAGTTTCGTCCTTTTGGACTCATCAGTAGGAAAACACATTC   240
FHVd-LR Oahu          GGTAGTAGCACTTTTGTTGAAGTTTCGTCCTTTTGGACTCATCAGTAGGAAAACACATTC   240
                      ************************************************************

Vd-LRNA Kauai short   CTAGACTTCGCGTGGGTGAGGGACAGCCGTCAGTGCGCCGTATATGCTATGAATATCTCA   300
FHVd-LR Oahu          CTAGACTTCGCGTGGGTGCGGGACAGCGGTCAGTGCGCCGTGCATGCTGTGTTTATCTCA   300
                      ****************** ******** *************  ***** **  *******

Vd-LRNA Kauai short   ATCAATTCATAGGTATACTGGACGAGTGCGGAGTTCCCAATACTCCCCTCTGAAGGTA     358
FHVd-LR Oahu          ATCAAAGCACAGATGTACTGGACGAGTGCCGAGTTCCCAATACTCCCCTCTGAAGGTA     358
                      *****  ** ** * ************** ****************************
```

Figure S3. Pairwise comparison between the short viroid-like RNA (Vd-LRNA) from Kauai island and FHVd-LR (master sequence) from Oahu island. The sequence identity between the two sequences is 96.09%. Positions with identical nucleotides are denoted by asterisks.
