# Supplementary material for: Novel Fig-Associated Viroid-Like RNAs Containing Hammerhead Ribozymes in Both Polarity Strands Identified by High-Throughput Sequencing
Source: Front Microbiol. 2020 Aug 18;11:1903. doi: 10.3389/fmicb.2020.01903 (PMC7461866; doi:10.3389/fmicb.2020.01903)
Supplement: FIGURE S4 — Northern blot hybridization with equalized full-length digoxigenine-labeled riboprobes for detecting FHVd-LR (+) or (−) strands (left and right panel, respectively). In each panel, lanes 1 and 2 correspond to equal amounts of dimeric FHVd-LR (+) and (−) transcripts, respectively. Positions of the FHVd-LR (+) and (−) linear monomeric forms (m) are indicated by an arrow. Position and size (in nt) of the RNAs used as molecular markers are reported on the left. [file Data_Sheet_4.PDF]

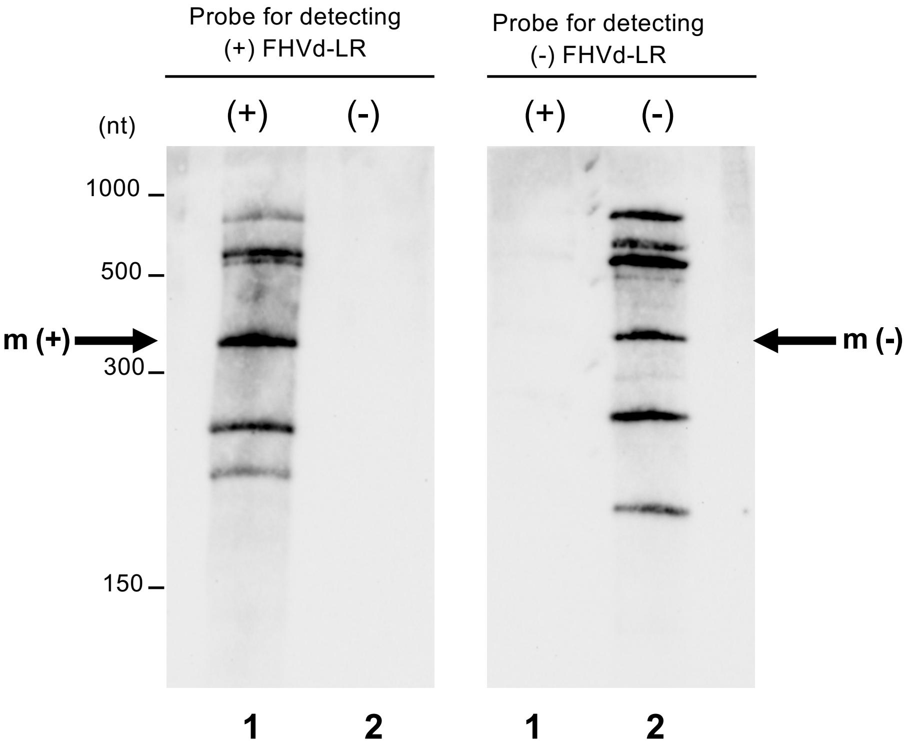

Figure S4. Northern-blot hybridization with equalized full-length digoxigenine-labeled riboprobes for detecting FHVd-LR (+) or (−) strands (left and right panel, respectively). In each panel, lanes 1 and 2 correspond to equal amounts of dimeric FHVd-LR (+) and (−) transcripts, respectively. Positions of the FHVd-LR (+) and (-) linear monomeric forms (m) are indicated by an arrow. Position and size (in nt) of the RNAs used as molecular markers are reported on the left.
