# Supplementary material for: Novel Fig-Associated Viroid-Like RNAs Containing Hammerhead Ribozymes in Both Polarity Strands Identified by High-Throughput Sequencing
Source: Front Microbiol. 2020 Aug 18;11:1903. doi: 10.3389/fmicb.2020.01903 (PMC7461866; doi:10.3389/fmicb.2020.01903)
Supplement: FIGURE S5 — (A) Schematic representation of plasmids containing head-to-tail dimeric constructs of FHVd-LR and of the products generated by in vitro transcription. Plasmids containing the dimeric sequence of FHVd-LR in opposite orientations were linearized with SalI or NcoI and transcribed with T7 or SP6 RNA polymerase, respectively. Transcription of these templates is expected to produce complete dimeric transcripts (D), fragments longer than a monomer (5′F-M and 3′F-M) generated by the self-cleavage of one hammerhead ribozyme (HRz), monomeric RNAs (M) and fragments (5′F and 3′F) generated by the self-cleavage of both HRzs. In green, plasmid sequences; in yellow, polymerase promoter; in blue, FHVd-LR sequence, with the arrows indicating the (+) orientation; arrowheads and scissors mark the positions of the self-cleavage sites. Numbers on the left of each RNA fragment indicate its expected size. (B) Analysis by PAGE of the in vitro transcription of plasmids containing (+) and (−) dimeric FHVd-LR cDNA; L1 and L2, RNA ladders with sizes indicated on the left and on the right, respectively; for other symbols see panel (A). [file Data_Sheet_5.PDF]

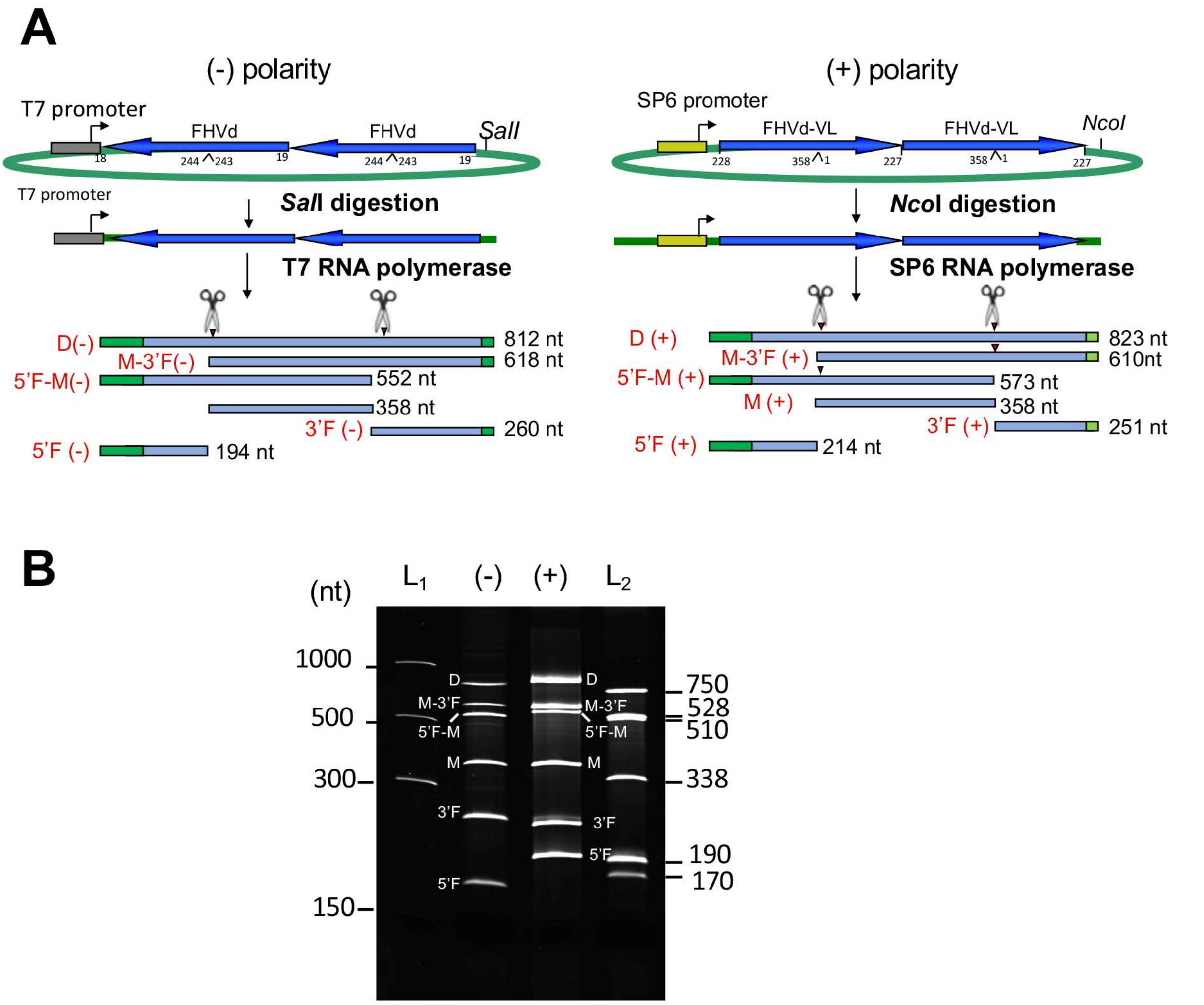

Figure S5. (A) Schematic representation of plasmids containing head-to-tail dimeric constructs of FHVd-LR and of the products generated by *in vitro* transcription. Plasmids containing the dimeric sequence of FHVd-LR in opposite orientations were linearized with *Sal*I or *Nco*I and transcribed with T7 or SP6 RNA polymerase, respectively. Transcription of these templates is expected to produce complete dimeric transcripts (D), fragments longer than a monomer (5'F-M and 3'F-M) generated by the self-cleavage of one hammerhead ribozyme (HRz), monomeric RNAs (M) and fragments (5'F and 3'F) generated by the self-cleavage of both HRzs. In green, plasmid sequences; in yellow, polymerase promoter; in blue, FHVd-LR sequence, with the arrows indicating the (+) oritentation; arrowheads and scissors mark the positions of the self-cleavage sites. Numbers on the left of each RNA fragment indicate its expected size. **(B)** Analysis by PAGE of the in vitro transcription of plasmids containing (+) and (-) dimeric FHVd-LR cDNA; L1 and L2, RNA ladders with sizes indicated on the left and on the right, respectively; for other symbols see panel A.
